# A Challenging Case of Dermatofibrosarcoma Protuberans (DFSP) Presenting With a Cone-Shaped Scalp Mass

**DOI:** 10.7759/cureus.97520

**Published:** 2025-11-22

**Authors:** Sooyie Choi, Yeon Woo Jung, Eunsung Cho, Byung Ho Oh

**Affiliations:** 1 Department of Dermatology, Galleria Dermatology Clinic, Seoul, KOR; 2 Department of Dermatology, Cutaneous Biology Research Institute, Yonsei University College of Medicine, Seoul, KOR

**Keywords:** dermatofibrosarcoma, mohs surgery, non-melanoma skin cancer, reconstruction, secondary intention healing

## Abstract

Dermatofibrosarcoma protuberans (DFSP) is a rare cutaneous malignancy characterized by extensive subclinical extension. Because of its locally destructive nature, most dermatologic surgeons perform Mohs micrographic surgery (MMS) to ensure negative margins. Undergoing multiple stages of MMS can be burdensome, and the subsequent reconstruction of massive cutaneous defects remains highly challenging.

Here, we report a case of a 44-year-old man with a recurrent DFSP of the scalp who developed a large surgical defect following slow MMS and was successfully managed with secondary intention healing (SIH). He presented with an adult fist-sized, flesh-colored mass on the occipital scalp, clinically recurring from DFSP locally excised 3 years earlier. Brain MRI revealed a 5.5 cm-sized enhancing mass without bone invasion. We performed four stages of slow MMS under local anesthesia, with partially excised basal periosteum. The final postoperative defect measured approximately 12 cm x 10 cm. Complete epithelialization was achieved within 19 weeks without postoperative complications, including infection.

SIH may offer a practical and effective reconstructive strategy for managing extensive post-excisional defects in non-melanoma skin cancers, including DFSP.

## Introduction

Dermatofibrosarcoma protuberans (DFSP) is a rare cutaneous malignancy characterized by marked subclinical extension and deep infiltration into the dermal and subcutaneous layers. Given its locally destructive nature, Mohs micrographic surgery (MMS) is widely regarded as the treatment of choice to ensure complete tumor clearance while preserving surrounding tissue [1]. A previous study reported a recurrent rate of 12.5% following conventional wide local excision, whereas no recurrences were observed after MMS during comparable follow-up periods [2].

However, the need for multiple stages of MMS and the subsequent requirement for complex surgical reconstruction of extensive cutaneous defects remain highly challenging. In anatomically constrained regions, such as the scalp, reconstruction can be particularly challenging due to limited tissue mobility and the inelastic nature of the galea. Secondary intention healing (SIH) has recently been recognized as a practical alternative in selected patients, providing satisfactory cosmetic outcomes without additional donor-site morbidity [3]. Here, we present a case of scalp DFSP successfully managed with MMS followed by SIH.

## Case presentation

**Figure 1:**
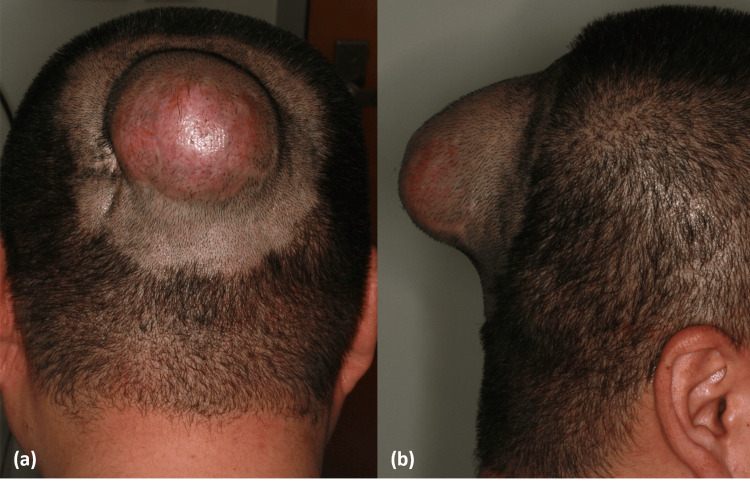
Preoperative clinical photographs of the occiput scalp mass (a) Posterior view showing a flesh-colored protruding lesion. (b) Lateral view of the same lesion.

**Figure 2:**
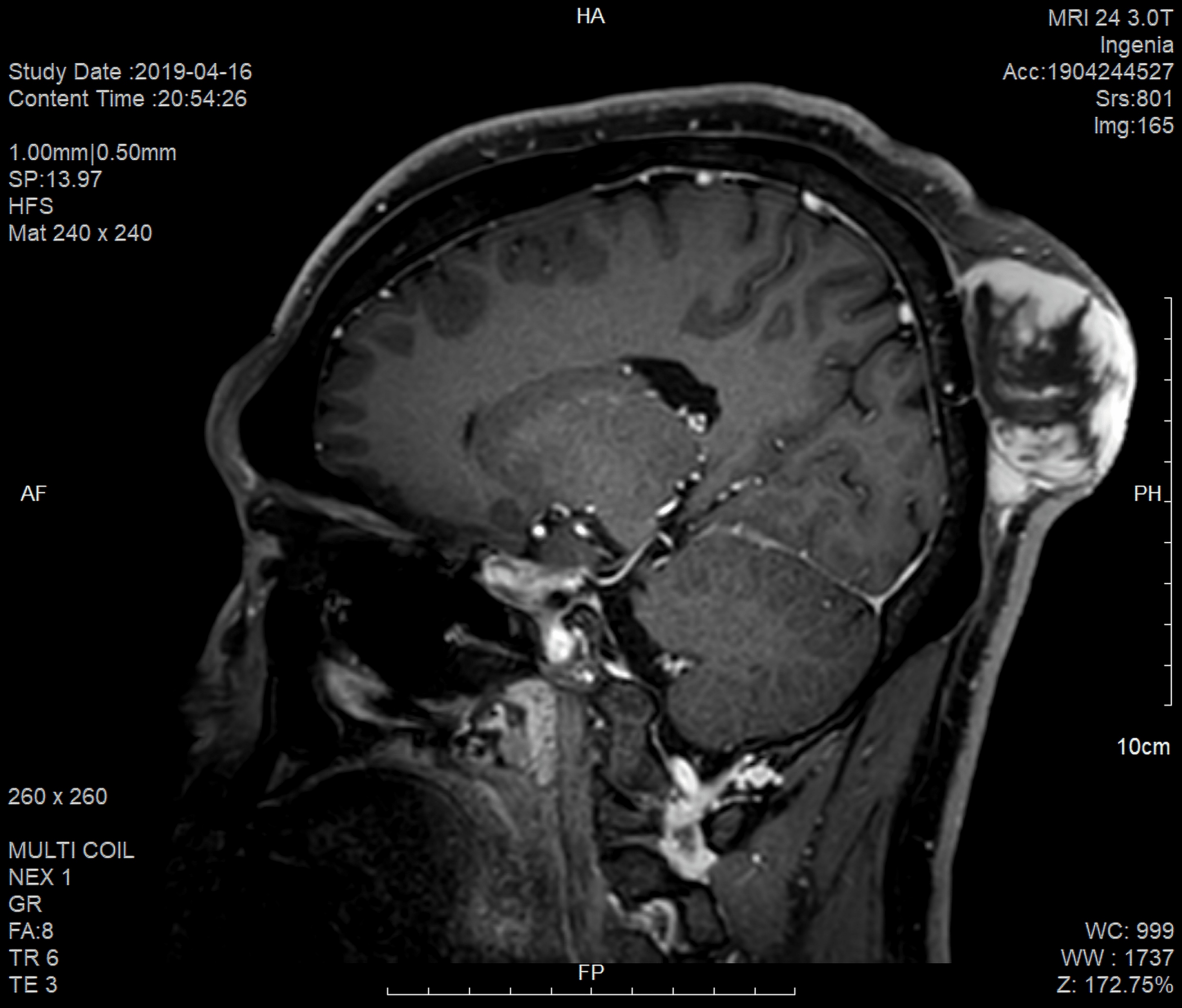
Preoperative magnetic resonance image revealing a large skin-to-periosteal tumor in the occipital area

**Figure 3:**
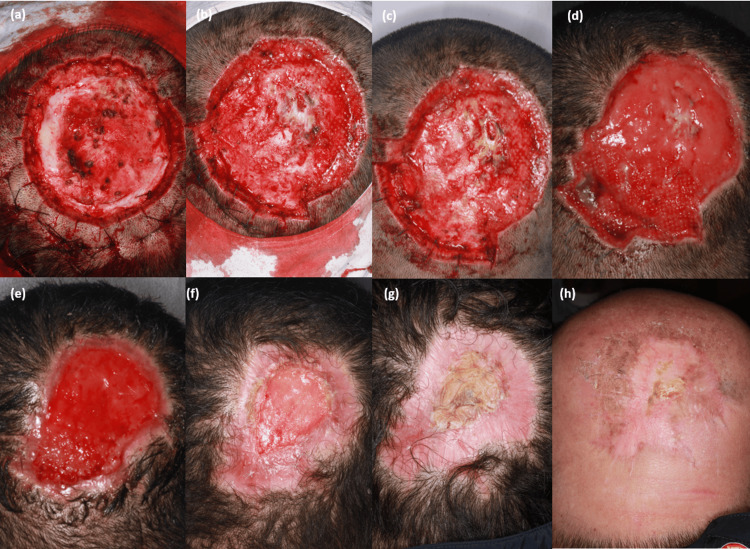
Intraoperative and postoperative clinical photographs (a) After the first stage of MMS. Interrupted sutures along the wound margin were placed solely for bleeding control. (b) After the second stage of MMS. (c) After the third stage of MMS. (d) Complete tumor removal after the fourth stage of MMS. (e) Postoperative week 8, showing full coverage of the exposed bone with granulation tissue and marginal epithelialization. (f) Postoperative week 12. (g) Complete epithelialization at postoperative week 19. (h) Postoperative month 8, following completion of adjuvant radiotherapy. The alopecic area corresponds to the radiation field and represents radiation-induced alopecia, not a complication of secondary-intention healing. MMS: Mohs micrographic surgery

A 44-year-old man presented with a baseball-sized, flesh-colored, protruding mass on the scalp that recurred from a DFSP, which had been locally excised three years earlier. Because of the underlying skull, the tumor expanded outward rather than invading deeply, resulting in a massive protrusion (Figure 1a, 1b). Magnetic resonance imaging demonstrated a large skin-to-periosteal tumor in the occipital area, extending outward without evidence of bone invasion (Figure 2). Under local anesthesia with a tumescent technique, multiple stages of slow MMS were performed to achieve negative margins in the inferior portion of the tumor (Figures 3a, 3b, 3c, 3d). After four stages of MMS, conducted over a 13-day period at three- to four-day intervals, complete removal of the tumor was achieved, along with partial removal of the periosteum (Figure 3d). Final histopathology revealed fibrosarcomatous DFSPs with focal necrosis. Immunohistochemical staining demonstrated focal loss of CD34 expression in the sarcomatous component and a Ki-67 index of approximately 10%, consistent with fibrosarcomatous transformation. During the staged MMS procedures, the patient remained hospitalized and received intravenous cefazolin as perioperative prophylaxis. After discharge, he completed a short course of oral amoxicillin-clavulanate, and topical mupirocin was applied during the early phase of SIH. One month later, a swab culture grew skin flora, for which an additional brief course of oral antibiotics was prescribed. During the 19-week healing period, the wound was managed with saline cleansing, a non-adherent siliconized contact layer, and an absorbent secondary dressing. Although negative-pressure wound therapy and skin substitutes were considered, they were ultimately not used due to patient refusal. Despite this, the wound remained clean throughout, with no clinically significant infection. The final postoperative defect measured 12 cm × 10 cm. By postoperative week eight, the previously exposed bone was fully covered with granulation tissue (Figure 3e), and by week 12, marginal epithelialization had progressed substantially (Figure 3f). Instead of primary closure or skin grafting, complete epithelialization was obtained by postoperative week 19 through SIH (Figure 3g). At 20 weeks postoperatively, the patient received adjuvant radiotherapy with a total dose of 60 Gy in 30 fractions and has remained recurrence-free for 20 months. The postoperative scar was cosmetically acceptable, with minimal contracture and no disfigurement (Figure 3h). During a five-year postoperative follow-up period, annual MRI examinations demonstrated no evidence of recurrence, including at the most recent assessment.

## Discussion

Several reconstructive techniques, including local or regional flap surgery, skin grafts, and SIH, are available for scalp defect reconstruction [4]. Rotation, advancement, or transposition flaps can be considered; however, these approaches require a reservoir of lax tissue that is not always available on the scalp. Both local flap surgery and skin grafting rely on highly vascularized donor and recipient sites to maximize engraftment success. Even when an adequate skin reservoir exists, skin grafting necessitates creation of an additional surgical wound at the donor site, and careful follow-up of the recipient site is required to prevent graft failure [5]. In particular, in cases of aggressive cutaneous malignancies where adjuvant radiotherapy is expected, most surgeons avoid skin grafting because of the increased risk of wound necrosis and graft failure [6].

By contrast, SIH can be performed without large-scale undermining and extensive use of a flap because any full-thickness skin wound heals by contraction and epithelialization following the natural course of wound healing. The timeline for granulation tissue formation and epithelialization varies depending on the defect size [7]. In this patient, however, the exposed bone was completely covered with granulation tissue within eight weeks, and full epithelialization was successfully achieved within 19 weeks. Despite concerns regarding potential complications of SIH, such as wound infection or delayed healing, the patient experienced no adverse events and was able to undergo adjuvant radiotherapy as scheduled. In our experience, SIH provided acceptable cosmetic outcomes, reduced operative time, and incurred significantly lower medical costs compared with local flap or skin graft procedures.

The procedural distinctions between MMS and slow MMS further inform surgical decision-making. MMS relies on intraoperative frozen-section analysis, enabling immediate microscopic margin evaluation and same-day sequential excision. In contrast, slow MMS employs staged excision with en-face permanent paraffin sections and delayed reconstruction, as documented in studies summarized by Meretsky and Schiuma [8]. This staged approach offers higher histologic accuracy in tumors where frozen-section interpretation is limited, and may be preferable in cases requiring permanent-section confirmation. Understanding these procedural distinctions helps contextualize margin-control strategies in DFSP and clarifies why slow MMS was selected in this case.

Recent studies have highlighted a renewed interest in SIH for scalp reconstruction, emphasizing its favorable cosmetic outcomes, simplicity, and patient satisfaction. In a case series of small-to-medium scalp defects, Daly et al. reported complete epithelialization within three to 12 weeks, no infections or hypergranulation, and uniformly excellent or good cosmetic outcomes [3]. Although their defects were smaller than ours, these data support SIH as a reliable reconstructive method on the scalp and help contextualize our patient’s 19-week epithelialization of a substantially larger (12 cm × 10 cm) defect without complications. Published data on SIH specifically for DFSP remain limited; however, in the largest review of scalp DFSP, Loss and Zeitouni noted that SIH or delayed closure may be appropriate when margin control is uncertain or recurrence risk is elevated, circumstances that closely align with our recurrent fibrosarcomatous DFSP case [1].

Although fibrosarcomatous DFSP represents a more aggressive histologic subtype compared with classic DFSP, DFSP as a whole remains a relatively slow-growing tumor. Large cohort data have shown that most recurrences occur years rather than months after treatment, with a median time to recurrence of approximately 35 months [9]. Earlier initiation of radiotherapy could theoretically have been achieved with flap or graft reconstruction; however, these techniques carry higher risks of wound breakdown or graft failure when combined with postoperative irradiation, particularly on the scalp and in defects involving partial periosteal removal. Given the absence of clinical or radiologic evidence of residual disease during the healing period, initiating adjuvant radiotherapy after complete epithelialization was unlikely to compromise oncologic safety in this case.

## Conclusions

Given the limited skin laxity and small tissue reservoir of the scalp, primary closure of extensive defects is particularly challenging. In our case, SIH offered several distinct advantages. It avoided the need for complex reconstruction, reduced operative morbidity, and allowed for close postoperative surveillance for local recurrence, which is an important consideration in DFSP management. SIH may be particularly advantageous for elderly or medically fragile patients or when adjuvant radiotherapy is planned, as it minimizes the risk of wound dehiscence and donor-site morbidity. Therefore, instead of complex local flaps or skin grafts, SIH represents a promising approach for managing large defects after tumor removal, especially in locally aggressive tumors such as DFSP.
